# Long Non-coding RNA Involved in the Pathophysiology of Atrial Fibrillation

**DOI:** 10.1007/s10557-023-07491-8

**Published:** 2023-09-13

**Authors:** Zikan Zhong, Xintao Li, Longzhe Gao, Xiaoyu Wu, Yutong Ye, Xiaoyu Zhang, Qingye Zeng, Changzuan Zhou, Xiaofeng Lu, Yong Wei, Yu Ding, Songwen Chen, Genqing Zhou, Juan Xu, Shaowen Liu

**Affiliations:** 1 Department of Cardiology, Shanghai General Hospital, Shanghai Jiao Tong University School of Medicine, Shanghai, China; 2 Department of Cardiology, The First Affiliated Hospital of Soochow University, Suzhou, Jiangsu China

**Keywords:** Atrial fibrillation, lncRNAs, ceRNA, Pathophysiological mechanism, lncRNAs-based therapy

## Abstract

**Background:**

Atrial fibrillation (AF) is a prevalent and chronic cardiovascular disorder associated with various pathophysiological alterations, including atrial electrical and structural remodeling, disrupted calcium handling, autonomic nervous system dysfunction, aberrant energy metabolism, and immune dysregulation. Emerging evidence suggests that long non-coding RNAs (lncRNAs) play a significant role in the pathogenesis of AF.

**Objective:**

This discussion aims to elucidate the involvement of AF-related lncRNAs, with a specific focus on their role as miRNA sponges that modulate crucial signaling pathways, contributing to the progression of AF. We also address current limitations in AF-related lncRNA research and explore potential future directions in this field. Additionally, we summarize feasible strategies and promising delivery systems for targeting lncRNAs in AF therapy.

**Conclusion:**

In conclusion, targeting AF-related lncRNAs holds substantial promise for future investigations and represents a potential therapeutic avenue for managing AF.

## Introduction

Atrial fibrillation (AF) is a prevalent and long-standing heart disease that has shown an increase in both prevalence and incidence, particularly among the aging population [1]. Moreover, AF is associated with various severe consequences such as stroke [2], heart failure (HF) [3], and myocardial infarction (MI) [4], which are major causes of mortality in AF patients. AF is a multifactorial cardiac arrhythmia that can be triggered by a range of factors. Structural heart diseases, including hypertension, coronary artery disease, and valvular heart disease, are commonly implicated as causes of AF [5]. Lifestyle choices such as excessive alcohol consumption, smoking, and obesity have been identified as modifiable risk factors for AF [6]. Furthermore, certain medical conditions, including thyroid disorders, chronic kidney disease, and sleep apnea, contribute to the development of AF [7–9]. Age and genetic predisposition also play crucial roles in increasing AF risk [10]. By elucidating these diverse factors, we aim to enhance our understanding of AF pathogenesis and facilitate the development of targeted prevention and treatment strategies. In fact, The pathogenesis of AF involves re-entry and ectopic activity, commonly referred to as the “substrate” and “trigger,” respectively, which play significant roles in the initiation and persistence of AF [11]. Multiple pathophysiological pathways, including electrical remodeling [12], Ca^2+^ handling abnormality [13], structural remodeling [14], and autonomic nervous system remodeling [15], have been deeply implicated in the onset and progression of AF [16]. More recently, scientists have started to focus on atrial energy metabolic remodeling and immunomodulation abnormality in the development of AF [17, 18]. However, the underlying molecular interaction pattern between these different pathways are not yet fully understood. Genome profiling analysis reveals that only approximately 2% of the DNA in the entire genome encodes proteins, while the remaining 90% or more consists of non-coding RNAs (ncRNAs). NcRNAs can be categorized into small ncRNAs and long non-coding RNAs (lncRNAs). LncRNAs are transcripts that exceed 200 bp in length, possess a 5' cap and a 3' polyadenylation tail, and lack open reading frames (ORF), indicating their inability to be translated into functional proteins. Consequently, lncRNAs were initially disregarded as “transcriptional noise” and received little attention. However, mounting evidence suggests that lncRNAs play crucial regulatory roles in gene expression [19] at various levels, including epigenetic [20, 21], transcriptional [22], and post-transcriptional [23, 24] regulation. Aberrant expression of lncRNAs has been implicated in the development of numerous cardiovascular diseases [25–29]. Recent studies have also demonstrated the involvement of competitive RNA interactions mediated by lncRNAs in the pathogenesis of AF (Table 1).

**Table 1:** AF-related LncRNAs mediate downstream pathways and their expressions in different samples and experimental models

AF-related lncRNA	Targeted miRNA	Target genes/signaling pathway	Function	Expression of lncRNA in samples	Effect for AF	Experimental model	Ref
FAM201A	miR-33a-3p	RAC3	predicted regulatory axis	downregulated in LAA,RAA,and LA-PV junction from AF	/	/	[30]
KCNQ1OT1	miR-223-3p	AMPK signaling pathway	predicted regulatory axis	upregulated in RAA from perAF	/	/	[31]
KCNQ1OT1	miR-384	CACNA1C	electrical remodeling	upregulated in the AF mice model(Ang-II-induced)	pro-AF effect	AF mice model(Ang-II-induced) and primary atrial cardiomyocyte from mice	[32]
TCONS_00075467	miR-328	CACNA1C	electrical remodeling	downregulated in RAA from the AF rabbit model(tachypacing-induced)	anti-AF effect	AF rabbit model(tachypacing-induced) and primary atrial cardiomyocytes from rabbit	[33]
MIAT	miR-135a	CACNA1C	electrical remodeling	downregulated in plasma samples from AF patients	anti-AF effect	AF mice model	[34]
TCONS_00106987	miR-26	KCNJ2	electrical remodeling	upregulated in the AF rabbit model(tachypacing-induced)	pro-AF effect	AF rabbit model(tachypacing-induced) and primary atrial cardiomyocyte from rabbit	[35]
MIAT	miRNA-133a-3p	CTGF/TGF-β1	structural remodeling	upregulated in peripheral blood leukocytes from AF patients and in right atrial tissue from AF rat model(tachypacing-induced)	pro-AF effect	AF rat model(tachypacing-induced)	[36]
MIAT	miR-485-5p	CXCL10	structural remodeling	upregulated in serum sample from AF patients	pro-AF effect	AF mice model(Ang-II-induced) and HL-1 cell	[37]
PVT1	miR-128-3p	Sp1/TGFβ1-Smad pathway	structural remodeling	upregulated in human atrial tissue	pro-AF effect	human CFs and AF mice model(Ang-II-induced)	[38]
H19	miR-29a/b-3p	VEGFA/TGF-β	structural remodeling	upregulated in serum sample from AF patients	pro-AF effect	rats CFs	[39]
LIPCAR	/	TGF-β/Smad pathway	structural remodeling	upregulated in atrial muscle tissue from AF patients	pro-AF effect	human CFs	[40]
GAS5	miR-21	PTEN/MMP-2	structural remodeling	dwonregulated in the AF mice model(isoproterenol-induced)	anti-AF effect	AF rat model(isoproterenol-induced) and primary CFs from rats	[41]
GAS5	/	ALK5/antifibrotic pathway	structural remodeling	downregulated in RAA from the AF patients	anti-AF effect	AC16 cell	[42]
LINC00472	miR-24	JP2/RyR2	Ca^2+^ handling abnormality	downregulated in serum and cardiac muscle tissue samples from AF patients	anti-AF effect	human cardiac myocytes(HCMs) and H9C2 cells	[43]
TCONS_00202959	/	/	autonomic nervous system remodeling	downregulated in the AF rat model	anti-AF effect	/	[44]
TCONS_00032546	/	CCND1-FGF19-FGF4-FGF3/MAPK pathway	autonomic nervous system remodeling	downregulated in the AF canine model	anti-AF effect	the AF canine model	[45]
TCONS_00026102	/	SLC25A4/NF-κB pathway	autonomic nervous system remodeling	downregulated in the AF canine model	pro-AF effect	the AF canine model	[45]
TCONS_00016478	/	PGC-1α/PPAR-γ pathway	energy metabolic remodeling	downregulated in RA tissue from the AF rabbit model(tachypacing-induced)	anti-AF effect	the AF rabbit model(tachypacing-induced)	[46]
NRON	miR-23a	macrophage polarization	immunomodulation abnormality	/	anti-AF effect	CFs and atrial myocyte from the AF mouse model(Ang-II-induced) and macrophage	[47]
PVT1	miR-145-5p	IL-16/macrophage polarization	immunomodulation abnormality	/	pro-AF effect	HCMs and human CFs and macrophage	[48]
XIST	miR-214-3p	Arl2/pyroptosis	immunomodulation abnormality	/	anti-AF effect	AF mice model(tachypacing-induced) and HL-1 cell and mice adipose tissue-derived mesenchymal stem cells(AMSCs)	[49]

**Fig. 1:**
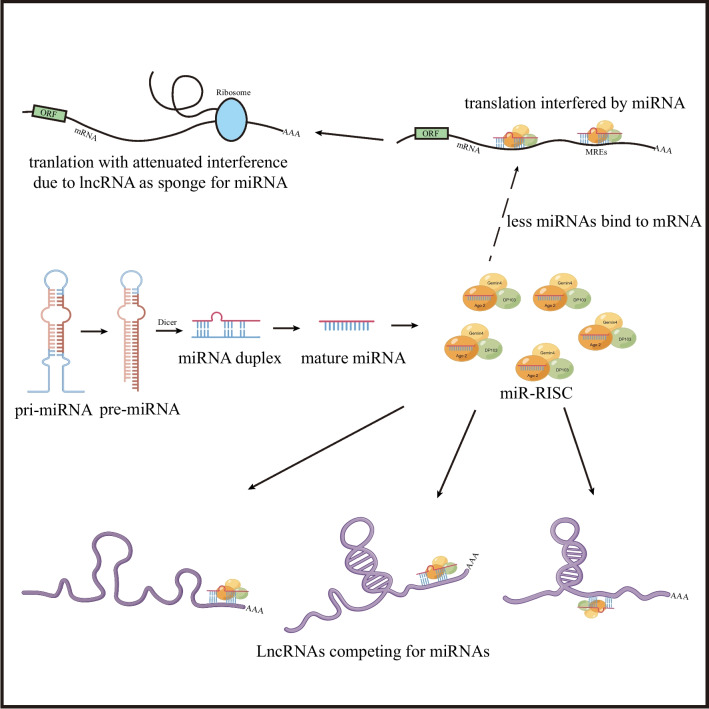
The ceRNA interaction pattern. MiRNAs form RISC that bind to mRNAs after processing and binding to related proteins to interfere with the translation process; LncRNAs can bind competitively with miRNAs to lower the inhibitory effect of miRNAs on mRNAs. Abbreviation: MREs, miRNA recognition elements; ORF, open reading frames

It has been demonstrated that miRNAs, short endogenous RNAs, bind to miRNA recognition elements (MREs) in protein-coding mRNAs, leading to the suppression of protein translation through miRNA-induced silencing complexes (miR-RISC) [50]. Conversely, lncRNAs possess an mRNA-like structure that enables them to bind to miRNAs [51]. Building upon this, Selmena et al. proposed the competitive endogenous RNA (ceRNA) theory [52]. According to this hypothesis, lncRNAs, through imperfect complementarity, compete with mRNA for miRNA binding sites. This interaction reduces the availability of miRNAs and attenuates their regulatory activity (Fig. 1). The involvement of ceRNAs in AF has been supported by several investigations [53–55]. For instance, overexpression of the lncRNA plasmacytoma variant translocation 1 (PVT1) has been shown to promote atrial fibrosis by modulating the miR-128-3p-SP1-TGF-β1-Smad axis in AF [38]. These findings suggest that dysregulated lncRNAs in atrial cells and tissues can influence the expression of key AF-related genes by sequestering miRNAs and thereby relieving their suppressive effects on downstream targets. It has been observed that many AF-related lncRNAs and dysregulated genes possess a high density of MREs, which were discovered shortly after the ceRNA hypothesis was proposed. This provides evidence for the existence of lncRNA-miRNA-mRNA interactions in AF. Furthermore, additional experimental data have confirmed the critical role of the lncRNA-miRNA-mRNA interaction network in various pathophysiological mechanisms of AF. In this review, we highlight studies focusing on the differential expression of AF-related lncRNAs, their significance in AF pathophysiology, and the current progress, challenges, and potential of lncRNAs in the diagnosis, prognosis, and therapy of AF.

## LncRNAs involved in the pathophysiology of AF

### LncRNA in eletricical remodeling

Atrial electrical remodeling (AER) is an early event in atrial remodeling and creates an environment conducive to electrophysiological abnormalities [16], thus facilitating the onset and maintenance of AF. AER primarily involves alterations in the atrial effective refractory period (AERP) and action potential duration (APD) [56, 57], which result from changes in cardiac ion channel expression. Notably, a reduction in I_CaL_ (L-type calcium current) and an upregulation of inward-rectifying K + current (I_K1_) are prominent features [58–61]. The significance of these abnormal currents in AF development has been extensively documented [57], leading to investigations of genes associated with these ion channels. For instance, miR-21 has been found to inhibit the expression of the α1c subunit of the calcium channel (CACNA1C), thereby reducing I_CaL_ and promoting a pro-AF effect [62]. Evidence suggests that dysregulation of ion channel-related genes, including potassium channels, plays a crucial role in AF susceptibility [63–66]. In recent years, numerous exploratory studies have implicated lncRNAs in ion channel-related genes during the AER processes induced by AF. Previous research has revealed that decreased I_CaL_ density and reduced APD contribute to the pathogenesis of electrical remodeling [12, 67]. Specifically, the CACNA1C gene encodes the cardiac L-type Ca^2+^ channel α1c subunit [68], while the potassium inwardly rectifying channel subfamily J member 2 (KCNJ2) gene encodes Kir2.1, which affects I_K1_. These findings highlight the essential involvement of CACNA1C and KCNJ2 in AF susceptibility [69].

#### CACNA1c-related lncRNA

##### TCONS_00075467/miR-328/CACNA1C

Li and colleagues [33] conducted a study to investigate the expression profile of lncRNAs in right atrial tissue using a rabbit model of AF induced by atrial tachypacing. Through high-throughput RNA-seq and qPCR validation, they observed the downregulation of a specific lncRNA, TCONS_00075467, along with reduced expression of CACNA1C. In vivo loss-of-function assays confirmed that knockdown of TCONS_00075467 led to a noticeable reduction in AERP and APD, indicating that inhibition of TCONS_00075467 increased AF inducibility. Applying the ceRNA theory and bioinformatics tools, they identified miR-328 as the binding miRNA, which was further confirmed by luciferase reporter assays. Co-transfection of RNAi-TCONS_00075467 lentiviruses and miR-328 inhibitors in both in vivo and in vitro experiments resulted in decreased expression of CACNA1C compared to the lenti-miR-328 inhibitor group. Rescue experiments demonstrated that TCONS_00075467 could stabilize the expression of CACNA1C by counteracting the inhibitory effects of miR-328, suggesting its role as a protective lncRNA in restricting or even reversing AER. Interestingly, miR-328 has been identified as a contributor to AF vulnerability by targeting CACNA1C and reducing I_CaL_ [70]. However, miR-328 and CACNA1C expression patterns differ between the left and right atria. Healthy human left atrial tissue exhibits lower CACNA1C expression and higher levels of miR-328 compared to the right atrium, and miR-328 expression displays age-dependent characteristics [71]. It is possible that TCONS_00075467 follows a similar age-dependent trend and exhibits inconsistent expression between the right and left atria.

##### KCNQ1OT1/miR-384/CACNA1C

Shen et al. [32] conducted a study that revealed the involvement of another lncRNA-miRNA pair in the modulation of CACNA1C expression. Interestingly, their findings contradicted the previous research mentioned. In their study, they observed the upregulation of a specific lncRNA, KCNQ1 opposite strand/antisense transcript 1 (KCNQ1OT1), and overexpression of CACNA1C in an Ang II-induced AF mouse model. They demonstrated that the knockdown of both KCNQ1OT1 and CACNA1C significantly improved AREP and interatrial conduction time (IACT), indicating that KCNQ1OT1 attenuates AER in Ang II-induced AF. Previous studies have shown that KCNQ1OT1 acts upstream of several miRNAs to modulate cardiomyocyte apoptosis in conditions such as HF [72], MI [73], and diabetic cardiomyopathy [74]. Using bioinformatic tools and rescue experiments, the authors identified a downstream miRNA, miR-384, inhibition of which could be reversed by the upregulation of KCNQ1OT1, leading to the abolition of CACNA1C inhibition. Through further functional experiments, they discovered an upstream regulator, YY1, a transcription factor whose expression correlated with KCNQ1OT1 and CACNA1C. Based on their findings, they postulated that the upregulation of the YY1-induced KCNQ1OT1/miR-384/CACNA1C pathway could facilitate abnormal electrical activity in the Ang II-induced AF mouse model.

##### MIAT/miR-135a/CACNA1c

Yao et al. [36] confirmed the involvement of the lncRNA myocardial infarction associated transcript (MIAT) as a new upstream profibrotic factor targeting TGF-β1, indicating its role in atrial structural remodeling. In another study, Zhao et al. [34] found that MIAT is also involved in AER. They discovered that MIAT, by acting as a miR-135a sponge, can influence the expression of target genes CACNA1c and I_CaL_, leading to cardiomyocytes with a APD. Based on their findings, the authors proposed a novel axis consisting of the lncRNA MIAT, miR-135a, and CACNA1c, which plays a pivotal role in AER.

#### IK1-related lncRNA

##### TCONS-00106987/miR-26/KCNJ2

Regarding I_K1_, several studies have shown that KCNJ2 levels impact atrial electrophysiology by affecting the encoding of the Kir2.1 channel and dysregulating I_K1_ [64, 65, 69, 75]. Additionally, miR-26 has been reported to inhibit KCNJ2 overexpression and increase I_K1_ [76]. In this context, Du et al. [35] discovered the upregulated differentially expressed lncRNA (DElncRNA) TCONS-00106987 in a rabbit model of AF. Gain- and loss-of-function assays conducted in the AF model revealed TCONS-00106987 as a pro-AF factor influencing AERP and AF incidence. Luciferase reporter assays and rescue experiments validated TCONS-00106987 as a ceRNA that sequesters miR-26, thereby modulating KCNJ2 expression. Consequently, Kir2.1 expression is upregulated, leading to a decrease in I_K1_ and facilitating AER. This study uncovered a novel regulatory lncRNA-miRNA-mRNA axis, the TCONS-00106987/miR-26/KCNJ2 axis, which participates in AER and contributes to AF development.

### LncRNA in structural remodeling

Atrial dilatation and tissue fibrosis are two characteristic features of structural remodeling that disrupt fiber bundle continuity and result in conduction disturbances [77]. Numerous studies have identified several profibrotic signaling pathways involved in the initiation and maintenance of AF [78], such as the TGF-β1/Smad signaling pathway [79]. Additionally, collagen synthesis and activation of cardiac fibroblasts (CFs) are molecular and cellular markers of atrial structural remodeling, representing complex multifactorial processes. Several novel LncRNAs have been discovered to interact with key factors implicated in structural remodeling, including transforming growth factor-β1 (TGF-β1), which is one of the most extensively studied components associated with this process. In addition to the TGF-β1/Smad signaling pathway, other profibrotic signaling pathways have also been identified. For instance, the renin–angiotensin–aldosterone system (RAAS)-related activated signaling pathway represents another common mechanism that promotes atrial fibrosis through the MAPKs/TGF-β1/TRAF6 axis [80]. Moreover, recent studies have highlighted the critical role of matrix metallopeptidases (MMPs) in the extracellular matrix. The balance between tissue inhibitors of metalloproteinases (TIMPs) and MMPs is crucial for collagen degradation, and dysregulation of this balance can lead to profibrotic stimulation, contributing to structural remodeling in AF [81].

#### LncRNA modulating TGF-β1/Smad typical pathway

##### PVT1/miR-128-3p/Sp1/TGF-β1/Smad pathway

Numerous studies have extensively investigated the role of the lncRNA PVT1 in various diseases, particularly in cancer, where it has been shown to have a sponging effect on miR-128-3p [82–84]. Recently, Cao et al. [38] conducted a pioneering study that revealed the regulatory function of the PVT1-miR-128-3p pair in AF. They observed higher expression levels of PVT1 in atrial muscle tissue of the AF group compared to the sinus rhythm group. Through further functional experiments conducted on human CFs, the authors demonstrated that PVT1 upregulates the expression of specificity protein 1 (Sp1) by attenuating the inhibitory effects of miR-128-3p, a mechanism that has also been reported in previous research [85]. The upregulated Sp1, acting as a transcription factor, binds to TGF-β1 and activates the TGF-β1/Smad pathway, thereby promoting cardiac fibrosis and collagen production. Silencing of PVT1 in an AF mouse model exhibited a mitigated effect on atrial dilatation induced by angiotensin II (Ang-II). Collectively, these findings highlight the novel therapeutic potential of targeting PVT1 in the treatment of AF.

##### LIPCAR/TGF-β/Smad pathway

A clinical trial [86] has revealed that patients with chronic HF exhibit elevated levels of lncRNA LIPCAR, indicating its potential as a clinical biomarker. LIPCAR has already been employed for the early detection of HF in patients post-acute MI [87]. Wang et al. [40] further elucidated the biological function of LIPCAR and confirmed its profibrotic effects. In their study, the authors analyzed atrial muscle tissue specimens from individuals with AF and those with sinus rhythm. They observed an upregulation of LIPCAR expression in the AF group, as well as increased levels of TGF-β1. Through in vitro transfection experiments involving RNAi-LIPCAR and LIPCAR overexpression plasmids, they demonstrated that the expression of proteins in the TGF-β1/Smad pathway correlated with the up- or downregulation of LIPCAR, along with collagen synthesis. These findings provide evidence that LIPCAR may contribute to structural remodeling by modulating the TGF-β1/Smad pathway.

#### LncRNA modulating non-typical pathway

##### GAS5/miR-21/PTEN/MMP-2

Collagen synthesis and degradation are crucial processes in cardiac fibrosis. MMP2, a biomarker associated with left atrial remodeling [88], has been identified as a downstream effector of the miR-21/phosphatase and tensin homolog (PTEN) signaling pathway [89–91]. As a gelatinase, MMP2 plays a role in ECM degradation, and its dysregulation results in collagen accumulation [92]. Additionally, miR-21 has been implicated in fibrosis in various organs, including the heart [93]. Furthermore, lncRNA growth arrest specific 5 (GAS5) has been shown in several studies to serve as an upstream regulatory molecule of miR-21, participating in the pathophysiological processes of various diseases, such as fibrosis [94], epithelial-mesenchymal transition (EMT) [95, 96], and apoptosis [97, 98]. Tao et al. [41] conducted a study using an isoproterenol-induced AF rat model, wherein they observed downregulation of GAS5 and increased collagen production in cardiac tissue. Moreover, the expression of miR-21 exhibited an inverse correlation with GAS5 levels in the samples, suggesting that GAS5 influences cardiac fibrosis by suppressing miR-21. Through rescue experiments, the authors elucidated the regulatory mechanism involving the PTEN/MMP2 signaling pathway. This study presents a novel modulating axis for reducing cardiac fibrosis and attenuating the development of AF, which holds potential as a therapeutic approach for AF.

##### MIAT/miR-485-5p/CXCL10

Recently, Chen et al. [37] discovered a novel function of MIAT: extracellular vesicles (EVs) containing MIAT that enhance structural remodeling by targeting the miR-485-5p/CXCL10 pathway. The C-X-C motif chemokine ligand 10 (CXCL10), a known pro-inflammatory chemokine, attracts various types of cells and is involved in multiple cellular processes [99]. Its profibrotic effect has also been observed in the liver [100]. In cardiovascular diseases, elevated expression of CXCL10 has been found in AF patients [101]. However, limited studies have focused on the role of CXCL10 in AF development. Chen et al. were the first to identify CXCL10 as a downstream factor of MIAT involved in AF. This study innovatively isolated and identified EVs from serum samples of AF patients and found increased expression of MIAT in these EVs, with the expression levels correlating with the severity of AF. The authors then investigated the role of EVs containing MIAT on HL-1 cells and an Ang-II-induced AF mice model, demonstrating that exosomal MIAT promotes fibrosis and structural remodeling. Moreover, database predictions identified the miR-485-5p/CXCL10 pathway as the downstream mechanism, and this novel lncRNA-miRNA pair was verified through luciferase reporter assays. Through further in vivo and in vitro functional experiments, the authors revealed that miR-485-5p, which downregulates CXCL10 expression, is reversed by overexpressing MIAT levels. However, this investigation did not establish a direct link between MIAT and the TGF-β1-related pathway, suggesting that CXCL10 may operate through an alternative fibrotic signaling pathway in AF.

#### LncRNA modulating unclear fibrotic-related signaling pathway

##### MIAT/miR-133a-3p/CTGF/TGF-β1/fibrotic pathway

Yao et al. [36] have confirmed that MIAT acts as an upstream profibrotic factor by regulating miR-133a-3p and influencing collagen deposition and the expression of connective tissue growth factor (CTGF)/TGF-β1. Initially, bioinformatic tools predicted miR-133a-3p as a potential binding miRNA for MIAT. Previous studies have reported miR-133a-3p to be a protective factor against cardiac fibrosis [102]. Subsequently, the authors observed similar findings in human leukocytes and an AF mice model, showing increased expression of MIAT and decreased expression of miR-133a-3p. The authors further demonstrated the pro-AF effects of MIAT and the anti-AF effects of miR-133a-3p through loss-of-function assays conducted in an AF rat model. Additionally, rescue experiments revealed that the electrophysiological changes and atrial fibrosis resulting from miR-133a-3p suppression could be counteracted by downregulating MIAT. Notably, the MIAT/miR-133a-3p/CTGF/TGF-β1 axis was also validated in a recent study investigating the effects of electroacupuncture therapy on myocardial fibrosis [103]. These findings propose a novel regulatory pathway involved in atrial structural remodeling in AF.

##### H19/miR-29a/b-3p/fibrotic pathway

Numerous reports have demonstrated the promoting effects of the miR-29/vascular endothelial growth factor A (VEGFA) axis on liver and pulmonary cell proliferation, as well as collagen synthesis [104, 105]. However, the lncRNA H19-miR-29a/b pair has exhibited regulatory roles in other diseases as well [106, 107]. Feng et al. [39] identified the involvement of lncRNA H19 upstream of miR-29a/b-3p in regulating atrial fibrosis in AF. They observed an upregulation of H19 and a decrease in miR-29a/b-3p levels in serum samples obtained from AF patients compared to controls. Importantly, the levels of H19 were found to increase with the progression of AF and the enlargement of the left atrium. These results suggest a correlation between the severity of AF and the expression of H19. Further functional experiments conducted on CFs in mice confirmed the interaction between H19 and miR-29a/b-3p. Moreover, the silencing of VEGFA or the inhibition of TGF-β significantly reversed the downregulation of miR-29a/b-3p-mediated CFs activation and collagen production. These experiments provided confirmation that H19 promotes myocardial fibrosis by modulating the miR-29a/b-3p-VEGFA/TGF-β axis.

##### GAS5/ALK5/fibrotic pathway

Another study [42] demonstrated that GAS5 suppresses CF proliferation in AF by inhibiting TGFβ type I receptor kinase (ALK5), a protein involved in TGFβ signaling and CF proliferation. In this investigation, the researchers observed a downregulation of the DElncRNA GAS5 and an increase in ALK5 levels in the RAA of AF patients. Subsequently, they conducted transfection experiments using GAS5 mimics and inhibitors in vitro and confirmed the inhibitory effects of GAS5 on CF proliferation and cardiac fibrosis through the downregulation of ALK5. It is worth noting that GAS5 has primarily been utilized as a clinical biomarker for predicting the progression and recurrence of AF [108].

### LncRNA in Ca^2+^ handling abnormality

It is well known that Ca^2+^-induced Ca^2+^ release serves as the foundation for myocardial contractility. The sarcoplasmic reticulum (SR) Ca^2+^ stores act as the source of Ca^2+^ and are regulated by two key “valves.” One valve is the ryanodine receptor 2 (RyR2), responsible for releasing Ca^2+^, while the other is the sarcoplasmic/endoplasmic reticulum Ca^2+^ ATPase 2 (SERCA2), responsible for uptaking Ca^2+^ [58]. During systole, a small amount of calcium entry triggers the opening of RyR2, resulting in cardiomyocyte contraction through the Ca^2+^-induced Ca^2+^ release mechanism. During diastole, Ca^2+^ is recycled through SERCA2 [109]. These two channels maintain a dynamic balance in maintaining Ca^2+^ homeostasis within the SR. Consequently, any abnormalities or dysregulation in the function of RyR2 or SERCA2 can lead to disrupted Ca^2+^ handling and arrhythmia [109].

For instance, the abnormal opening of a significant amount of RyR2 during diastole can trigger local inter-RyR Ca^2+^-induced Ca^2+^ release, known as spontaneous SR Ca^2+^-release events (SCaEs). Additionally, the frequent spark of Ca^2+^ initiated by L-type Ca^2+^ channels, known as triggered Ca^2+^ waves (TCWs), during diastole can lead to abnormal activity in associated ion channels such as the Na^+^/Ca^2+^ exchanger (NCX) current. Both SCaEs and TCWs can cause ectopic activity, such as delayed afterdepolarizations (DADs) [110, 111]. On the other hand, reduced activity of SERCA2 can impair proper Ca^2+^ recycling and result in unsustainable Ca^2+^ loading within the SR. This leads to increased cytosolic Ca^2+^ levels, which can also trigger abnormal activity and initiate AF. The imbalance between leak and uptake further exacerbates the development of AF [112].

#### RyR2 channel-related lncRNA

##### LncRNA LINC00472/miR-24/JP2/RyR2

RyR2 plays a crucial role in the Ca^2+^ leak pathway, enabling a significantly larger release of Ca^2+^ from SR storage. Previous studies have demonstrated that gain-of-function mutations in RyR2 increase AF inducibility [113]. Furthermore, AF development has been associated with RyR2 hyperphosphorylation and the abnormal release of a large amount of Ca^2+^ into the cytoplasm [114]. Junctophilin-2 (JP2) functions as a regulatory molecule by directly binding to RyR2, modulating its activity, and participating in SR electrical coupling. This interaction contributes to the stabilization of RyR2 expression [115, 116]. In atrial tissue from AF patients, Wang et al. [43] identified a DElncRNA called LINC00472 and discovered a negative correlation between LINC00472 and miR-24. Interestingly, LINC00472 has been shown to exert its effects by binding to miR-24 in various diseases [117], while miR-24 has been demonstrated to modulate JP2 in cardiomyocytes [118]. In their study, the authors confirmed the binding relationship between LINC00472 and miR-24-3p through luciferase reporter assays. Furthermore, co-transfection of an overexpressed LINC00472 plasmid and an anti-miR-24-3p plasmid resulted in higher levels of JP2 and RyR2 compared to the group transfected with only the LINC00472 plasmid. These findings establish the existence of a regulatory axis, LINC00472/miR-24/JP2/RyR2, which plays a role in Ca^2+^ handling and the development of AF.

#### SERCA2a channel-related lncRNA

No lncRNA has been directly linked to the dysregulation of SERCA2a in AF. However, there are potential lncRNAs that may modulate SERCA2a under certain pathological conditions, offering insights into AF development. Knockdown of lncRNA zinc finger antisense 1 (ZFAS1) has demonstrated a restorative function in several cardiovascular disease models [119–121]. Additionally, ZFAS1 has been proposed as a biomarker for MI [122]. Recent studies have identified ZFAS1 as a suppressor of SERCA2a expression [123, 124]. Both studies demonstrated that the binding of ZFAS1 to SERCA2a plays a critical role in MI, promoting cytosolic Ca^2+^ overload and contractile abnormalities. It is hypothesized that ZFAS1 may contribute to an inward Na^+^/Ca^2+^ exchanger (NCX) current and delayed afterdepolarizations (DADs), potentially making it a new candidate involved in AF development [123, 124]. Similarly, the lncRNA DACH1 was found to promote SERCA2a degradation through ubiquitination, leading to impaired Ca^2+^ homeostasis [125]. Furthermore, another study discovered that the lncRNA MIAT also plays a critical role in exacerbating Ca^2+^ handling abnormalities through the downregulation of RyR2 and SERCA2a [126]. This study proposed a novel regulatory mechanism of MIAT in AF by disrupting Ca^2+^ homeostasis in the atrium.

### LncRNA in autonomic nervous system remodeling(ANSR)

Increasing evidence indicates the growing significance of autonomic nervous system remodeling (ANSR) in the development of AF [127]. A randomized clinical trial confirmed that combining autonomic plexus ablation with pulmonary vein isolation (PVI) increased the success rate of ablation, highlighting the involvement of ANSR in AF development [128]. Several studies have shown that excessive adrenergic activation contributes to impaired atrial neural activity, ultimately leading to the initiation and progression of AF [129, 130]. Nerve sprouting, a characteristic of ANSR, has been observed in canine models induced by tachypacing, along with greater nerve density in atrial tissue [131]. Sympathovagal coactivation is now recognized as a mechanism underlying AF development, rather than a singular disorder of sympathetic or vagal nerves [132, 133]. The specific innervation pattern of the cardiac autonomic nervous system may vary among different AF patients. Cholinergic induction has been used as an effective method to create animal models of AF, while sympathetic hyperinnervation is associated with the onset and persistence of AF. Although both activations play a role in AF development, it appears that the combined activation and ordered imbalance between the two are the most likely mechanisms of ANSR [45]. In addition, Jiang et al. [134] conducted bioinformatic analysis on the lncRNA expression profile of the right anterior ganglion plexus in dogs with AF and identified DElncRNAs, suggesting the involvement of lncRNAs in ANSR. Subsequent evidence indicates that these DElncRNAs may mediate ANSR and potentially serve as targets for AF diagnosis and treatment.

#### LncRNA TCONS_00202959

Zhao et al. [44] observed a downregulated expression of lncRNA TCONS_00202959 in an AF rat model, which was accompanied by a reduction in APD and a decrease in AF induction. Remarkably, when the expression of TCONS_00202959 was increased in vivo, the electrophysiological ANS-related indexes demonstrated a recovery of neurological function. Additionally, the levels of tyrosine hydroxylase (TH) and choline acetyltransferase (CHAT), biomarkers of sympathetic and parasympathetic activity, were measured. The AF group exhibited abnormal levels of TH and CHAT, whereas the treatment group with elevated TCONS_00202959 showed a restoration of these dysregulated indicators. These results suggest a protective function of TCONS_00202959 in AF development. Based on these findings, the authors hypothesized that TCONS_00202959 may act as an upstream regulatory factor, exerting an influence on the development of AF by suppressing ANSR.

#### TCONS_00032546/CCND1-FGF19-FGF4-FGF3/MAPK pathway and TCONS_00026102/SLC25A4/NF-κB pathway

Wang et al. [45] conducted a study using an AF dog model to investigate the transcriptomes of lncRNAs in the anterior right atrial fat pad. They identified two downregulated lncRNAs, namely TCONS_00032546 and TCONS_00026102, which exhibited distinct functions. Loss-of-function experiments demonstrated that TCONS_00032546 plays an anti-AF role, whereas inhibition of TCONS_00026102 reduced AF inducibility. Moreover, the authors assessed nerve density and observed an increase in nerve density upon downregulation of TCONS_00032546, whereas the TCONS_00026102 knockdown group showed a decrease in nerve density. Additionally, employing the concept of lncRNA-mediated regulation of neighboring genes, Wang and colleagues predicted the potential downstream pathways associated with TCONS_00032546 and TCONS_00026102, which may contribute to ANSR. Consequently, they proposed two regulatory axes, namely the TCONS_00032546/CCND1-FGF19-FGF4-FGF3/MAPK pathway and TCONS_00026102/SLC25A4/NF-κB pathway, which are involved in ANSR and AF development.

### LncRNA in energy metabolism remodeling

Numerous researchers have extensively investigated the involvement of lncRNAs in energy metabolism in heart diseases. For instance, Liu et al. [135] identified a novel lncRNA called LncHrt, which is highly expressed in cardiomyocytes. Functional experiments further demonstrated that LncHrt plays a cardioprotective role by restoring the energy metabolic system in ischemic heart diseases. Similarly, previous studies have highlighted the significance of disrupted metabolic homeostasis in the atrium, which contributes to the pathophysiology of AF [136, 137]. This atrial energy metabolic remodeling encompasses various aspects, including mitochondrial dysfunction [17], impairment of high-energy phosphate stores [136], dysregulation of critical enzymes [138], excessive accumulation of metabolites [139], and other mechanisms that remain unclear. Importantly, over the past few decades, several studies have identified specific lncRNAs that act as regulators within these signaling pathways, exerting functional roles in the development of AF [140, 141].

#### AF-related lncRNA in PPARs/sirtuin/PGC-1 signaling pathway

Peroxisome proliferator-activated receptors (PPARs) are highly expressed in atrial tissue and play a crucial role in stabilizing lipid metabolism [142]. Multiple studies have demonstrated that disruption of PPARs leads to cardiac dysfunction by promoting fatty acid oxidation (FAO) and lipid accumulation [143, 144]. However, limited research has focused on the function of PPARs in AF [145, 146]. PPARγ coactivator-1 s (PGC-1 s) have been identified as binding partners of PPARs, and together they coactivate gene expression to stabilize lipid metabolism and lipoprotein levels, thus preventing metabolic energy impairment [147, 148]. Another essential regulator within this pathway is sirtuin1, which acts as a deacetylase and modulates the function of PGC-1ɑ in energy metabolism [149, 150]. Liu et al. [151] identified a critical pathway, namely the PPAR-ɑ/sirtuin1/PGC-1ɑ pathway, implicated in AF metabolism remodeling. Their study revealed significant suppression of key metabolic-related proteins in this pathway, as well as aberrant accumulation of metabolites in AF patients and models. These findings suggest that targeting the PPAR-ɑ/sirtuin1/PGC-1ɑ axis could be a novel therapeutic approach for AF. Although the biological effects of PPARs/sirtuin1/PGC-1ɑ signaling pathway-related lncRNAs have been extensively investigated in other diseases [152–154], their roles in the pathogenesis of AF remain largely unknown.

##### LncRNA TCONS_00016478/PGC-1α/PPAR-γ pathway

Using nanosensor technology, Jiang et al. [46] identified a downregulated DElncRNA, TCONS_00016478, in an AF rabbit model induced by tachypacing. They established a meaningful association between this lncRNA and atrial energy metabolic remodeling. Through informatic prediction and subsequent loss-of-function experiments, the authors determined that the PGC-1α/PPAR-γ axis serves as a downstream signaling pathway regulated by TCONS_00016478. Knockdown of TCONS_00016478 resulted in a significant reduction in the expression of PPARγ, PGC-1α, as well as other metabolism-related enzymes and proteins. This dysregulation of atrial energy metabolism underscores the critical role of TCONS_00016478 in AF. These studies represent the first identification of an lncRNA involved in AF energy metabolic remodeling through modulation of the PGC-1α/PPAR-γ signaling pathway.

##### Other potential lncRNAs

Furthermore, Niu et al. [155] identified lncRNA OIP5 antisense RNA 1 (Oip5-as1) as a miR-29a decoy that inhibits mitochondrial dysfunction and oxidative stress, thereby promoting the SIRT1/PGC-1 pathway. This finding suggests that Oip5-as1 may serve as a protective mechanism in reversing AF. Similarly, another study focused on SIRT1 has confirmed the cardiac reparative effect of lncRNA antisense non-coding RNA in the INK4 locus (ANRIL), which attenuates oxidative stress by acting as a sponge for miR-181a [156]. However, these findings have not been validated in AF models or patients. Therefore, the precise effects of Oip5-as1, ANRIL, and the functional networks involving lncRNAs in heart metabolism remodeling and their impact on AF still require further investigation.

### LncRNA in immunomodulation abnormality

Recently, numerous findings have demonstrated the significant involvement of immunomodulation abnormalities in the initiation and maintenance of AF [157–159]. For instance, treatments incorporating anti-inflammatory agents such as steroids or vitamin C have shown the ability to attenuate AF progression [160, 161]. Additionally, the interaction between macrophages and atrial myocytes has emerged as a novel molecular mechanism underlying AF. Sun et al. [162] have elucidated that AF promotes the polarization of pro-inflammatory macrophages, known as M1 phenotype macrophages, while inhibiting the anti-inflammatory M2 phenotype macrophages [163]. Conversely, enhancing the M1 phenotype and inhibiting the M2 phenotype has been shown to facilitate cardiac fibrosis and AF progression [164]. Moreover, another study has revealed immune cell infiltration in atrial tissue, specifically the presence of inflammatory CD3-positive T cells, further emphasizing the crucial role of aberrant immunological activity in AF [165].

Several researchers have conducted bioinformatics analyses using the GEO database and identified the significance of lncRNA-related immune cell infiltration in the pathogenesis of AF [157, 166]. Xie et al. [157] performed differential expression analysis and discovered an adverse association between LINC00844 and resting dendritic cells. These findings suggest that DElncRNAs may contribute to AF susceptibility through immunological regulatory networks. Furthermore, gene set variation analysis (GSVA) predicted the involvement of toll-like receptor (TLR) and IL-7-mediated signaling pathways in the onset and progression of AF. Notably, both pathways have been found to play essential roles in cardiovascular diseases, respectively, through the innate immune system and expansion of T lymphocytes [167–169].

#### Macrophage M1/M2 polarization-related lncRNA

##### NRON/miR-23a/macrophage M2-type polarization

The non-coding repressor of NFAT (NRON), previously implicated in cell proliferation [170], has recently been identified as a critical factor associated with macrophage M1/M2 polarization [171]. In a study by Li et al. [47], it was demonstrated that exosomal miR-23a secreted by Ang-II-induced atrial myocytes can inhibit M2-type polarization of macrophages, and NRON plays a suppressive role in regulating exosomal miR-23a levels, as confirmed by gain-of-function experiments. Further, functional experiments revealed that NRON promotes M2-type polarization and attenuates atrial fibrosis by inhibiting exosomal miR-23a. This study unveiled a novel regulatory mechanism for suppressing cardiac fibrosis, suggesting NRON as a potential therapeutic target for AF. Interestingly, NRON achieves the downregulation of miR-23a by suppressing NFATc3, a transcription factor of miR-23a, rather than functioning as a ceRNA.

##### PVT1/miR-145-5p/IL-16/macrophage M1-type polarization

The pro-fibrotic role of PVT1 in structural remodeling has been extensively discussed [38]. The same authors have further investigated the involvement of PVT1 in macrophage polarization [48]. In their study, the authors focused on the impact of exosomal PVT1, secreted by atrial myocytes, on macrophages. Initially, they successfully isolated exosomal PVT1 from Ang-II-induced atrial myocytes and confirmed its significantly elevated expression, as well as its role in promoting M1-type macrophage polarization. Through the utilization of prediction tools and luciferase reporter gene assays, they elucidated the downstream pathway of PVT1 involving miR-145-5p/IL-16. Notably, the PVT1-miR-145-5p pair has demonstrated functional relevance in multiple diseases [172–174]. Furthermore, rescue experiments verified that exosomal PVT1 enhances M1-type macrophage polarization by counteracting the inhibitory effect of miR-145-5p on IL-16, thereby promoting atrial fibrosis and exacerbating AF. These findings highlight the pivotal role of exosomal PVT1 in abnormal immunomodulation and the development of AF, presenting a potential novel therapeutic approach for AF.

#### Pyroptosis-related lncRNA

Pyroptosis, a form of programmed cell death closely associated with the inflammatory response, has garnered considerable attention from scientists in recent years [175]. An essential component of pyroptosis is NLRP3, whose activation can trigger downstream inflammatory cascades, resulting in the release of substantial amounts of inflammatory factors, ultimately leading to cell swelling and pyroptosis [176]. Moreover, pyroptosis has been recognized as a significant contributor to various cardiovascular disorders, including MI [177] and diabetic cardiomyopathy [178]. While some studies have suggested the importance of NLRP3 in the pathophysiology of AF [179], the specific lncRNAs related to pyroptosis have not yet been associated with AF.

##### XIST/miR-214-3p/Arl2/pyroptosis

Several studies have reported the potential protective function of X-inactive specific transcript (XIST), a lncRNA, against cardiovascular disorders, including MI [180, 181]. Recently, it has been discovered that exosomal XIST can promote pyroptosis by acting as a sponge for specific miRNAs and increasing the expression of their target genes [182]. In an innovative study, Yan et al. [49] isolated EVs secreted from adipose tissue-derived mesenchymal stem cells (AMSCs) in mice and identified the presence of XIST within these EVs. They demonstrated that the overexpression of exosomal XIST in an AF mouse model and HL-1 cells had a functional role in repressing the NLRP3 inflammasome and pyroptosis. Through bioinformatic prediction and luciferase reporter gene assays, they confirmed the miR-214-3p/ADP ribosylation factor like GTPase 2 (Arl2) pathway as the downstream target of XIST. Rescue experiments further confirmed that exosomal XIST could enhance Arl2 expression and suppress pyroptosis by counteracting the inhibitory effect of miR-214-3p in both in vivo and in vitro settings. These findings suggest a novel avenue for future investigations into the role of exosomal lncRNAs and pyroptosis in AF.

## Other potential DElncRNAs in AF patients

### LncRNA interaction network construction by bioinformatic analysis

With the advancements in bioinformatics, a substantial amount of data on AF has been obtained in the past decade through biospecimen sampling. Xie et al. [157] conducted microarray analysis on four integrated GEO datasets to assess lncRNA expression, identifying six DElncRNAs and 45 differentially expressed mRNAs (DEmRNAs) in AF patients. They also constructed lncRNA interaction networks and found that DElncRNAs were involved in immune response abnormalities as AF progressed. Qian and colleagues constructed an AF-related lncRNA-mRNA network using the GEO database, identifying 107 lncRNA-mRNA regulatory pairs, of which 91 were gain-of-function pairs and 16 were loss-of-function pairs. Furthermore, they observed that the downstream pathways of these pairs contribute to the electrical and structural remodeling of AF, suggesting a crucial role of these potential lncRNA-miRNA-mRNA networks in AF development [53]. Wu et al. collected atrial tissue samples from seven AF patients and controls, and through RNA sequencing, they identified DElncRNAs and differentially expressed circular RNAs (DEcircRNAs). After validation by RT-PCR, they constructed lncRNA-miRNA-mRNA and circRNA-miRNA-mRNA maps. Moreover, GO and KEGG enrichment pathway analysis identified eight molecular mechanisms related to these networks, such as the MAPK signaling pathway [183]. Ruan et al. [184] collected peripheral blood monocytes from 20 pairs of AF patients and controls and used gene chip technology to detect DElncRNAs in AF and sinus rhythm patients. They discovered 19 DElncRNAs, with six upregulated and 13 downregulated. GO and KEGG analysis indicated that several enriched DElncRNAs were linked to Ca^2+^-related and NF-κB signaling pathways, suggesting their importance in the pathogenesis of AF. Through the creation of an lncRNA-mRNA interaction map, the authors highlighted lncRNA HNRNPU-AS1 as the most AF-related lncRNA in the networks, suggesting its significant role in AF development. In recent years, adipose tissue depots have been implicated in AF vulnerability [185], yet only a limited number of studies have investigated lncRNAs in epicardial adipose tissue (EAT). Zhao et al. [186] took a novel approach by focusing on DElncRNAs in EAT and identified 57 DElncRNAs (17 upregulated and 40 downregulated) in the AF group compared to the sinus rhythm group.

### Potential predicted lncRNA-miRNA-mRNA axis

#### FAM201A/miR-33a-3p/RAC3

Chen et al. conducted bioinformatic analysis on appendage tissue obtained from AF patients who underwent cardiac surgery. Their study resulted in the construction of a ceRNA network consisting of five DElncRNAs, ten differentially expressed miRNAs (DEmiRNAs), and 21 DEmRNAs [30]. Using weighted gene co-expression network analysis (WGCNA), the authors proposed a novel regulatory pathway involving family with sequence similarity 201 member A (FAM201)/miR-33a-3p/Rac family small GTPase 3 (RAC3), which may contribute to the pathogenesis of AF by promoting autophagy. Autophagy has been implicated in AF development, as increased autophagy of L-type calcium channels leads to a reduced APD and downregulation of I_CaL_ [187]. The authors hypothesized that the downregulation of FAM201A resulted in the upregulation of RAC3, which could enhance autophagy of L-type calcium channels and increase susceptibility to AF.

#### KCNQ1OT1/miR-223-3p/AMPK signaling pathway

In a recent study, Dai et al. conducted a microarray analysis on right atrial appendage (RAA) tissue obtained from patients with persistent AF who underwent open-chest surgery. They identified several DEmiRNAs and specifically focused on the upregulated miRNA miR-223-3p for further investigation. This miRNA has previously been associated with AF in other bioinformatic analyses [31, 188]. Furthermore, miR-223-3p has been shown to play a role in myocardial fibrosis and exert anti-inflammatory effects in myocardial ischemia/reperfusion injury [189, 190]. Based on these findings, Dai and colleagues identified predicted target genes of miR-223-3p and confirmed their enrichment in the AMPK signaling pathway. Using bioinformatic tools and luciferase reporter gene assays, they also identified the interaction between the lncRNA KCNQ1OT1 and miR-223-3p. KCNQ1OT1, previously recognized as an AF-related lncRNA contributing to electrical remodeling in AF, was thus implicated in a potential regulatory axis involving miR-223-3p and the AMPK signaling pathway [32].

Collectively, the results of this bioinformatic analysis and the construction of the lncRNA-miRNA-mRNA interaction map provide further insights into the pathophysiological effects of lncRNAs in AF. These findings suggest that lncRNAs could be explored as potential clinical markers for diagnosis, prognosis, and therapeutic targeting. However, it is important to note that bioinformatics studies can only establish correlations between DElncRNAs and the occurrence and progression of AF. Causal relationships have not been established, emphasizing the need for further validation in future studies.

## Discussion

### Limitation of lncRNA studies

The research methods employed for studying lncRNAs and AF encompass various approaches. Bioinformatics analysis involves the analysis of publicly available gene expression data, such as microarrays and RNA sequencing data, to perform differential expression analysis, construct gene networks, and conduct pathway analysis, aiming to explore the potential roles of lncRNAs in AF. Subsequently, gene expression experiments are conducted by collecting tissue samples or peripheral blood cells from AF patients and normal control groups to identify DElncRNAs and their associated genes. Functional validation experiments are then employed to evaluate the impact and regulation of lncRNAs on the pathogenesis of AF. These experiments may involve overexpressing or inhibiting lncRNAs, or employing small molecule drugs targeting specific lncRNAs in cell-based studies and animal models. In vitro studies, utilizing cell lines or cultured cardiomyocytes, have demonstrated that certain lncRNAs, when overexpressed or inhibited, can affect downstream signaling pathways related to ion channels, cell apoptosis, cell proliferation, and inflammatory responses, leading to cardiac electrophysiological and structural remodeling in AF. In vivo studies, conducted using tissue samples from AF patients and animal models, have revealed altered expression levels of specific lncRNAs, which are associated with cardiac electrophysiological abnormalities, fibrosis, inflammatory responses, and autophagy processes. Moreover, intricate regulatory networks involving lncRNAs, miRNAs, and mRNAs have been identified, participating in the regulation of gene expression and activation or inhibition of signaling pathways related to AF. In summary, through the integration of bioinformatics analysis, in vitro cell experiments, and in vivo studies, researchers have identified a series of lncRNAs associated with AF. However, it is important to note that most of these findings represent preliminary evidence or shallow associative studies, requiring further validation and in-depth investigation to understand the specific functions and regulatory mechanisms of lncRNAs in AF, as well as their potential applications in diagnosis, prognosis, and treatment.

Based on the previous summary, it is evident that the pathophysiological mechanisms underlying AF development are interconnected rather than independent, exhibiting a causal relationship. For instance, disruptions in calcium homeostasis have an impact on mitochondrial membrane potentials, leading to metabolic energy remodeling [17]. Additionally, abnormal immunomodulation often triggers fibrosis-related pathways as downstream cascades [18]. Furthermore, remodeling of the autonomic nervous system results in a functional counterbalance of various channel currents, which contributes to electrical remodeling [191]. These multifactorial mechanisms contribute to the complexity of AF. However, it is important to note that lncRNAs play a crucial role not only as miRNA sponges in the cytoplasm but also in the nucleus and extracellular compartments [192]. LncRNAs can also act as downstream components of miRNA-mRNA regulatory networks, potentially playing an arrhythmogenic role [193]. Exploring these diverse functional patterns of lncRNAs in the context of AF is an area that requires further investigation.

Furthermore, the previous summary highlights that different lncRNA-miRNA axes can converge on the same downstream target gene, such as CACNA1C and SERCA2, regulating distinct regulatory endpoints [32, 34, 43, 123, 125]. Similarly, a single lncRNA can target multiple pathways. For instance, both PVT1 and GAS5 have been found to regulate different downstream signaling pathways involved in AF development [38, 41, 42, 48]. Moreover, MIAT has been shown to exert AF-related effects on electrical and structural remodeling [34, 36]. These observations suggest that a single lncRNA may simultaneously participate in multiple AF developmental mechanisms. However, the specific spatiotemporal expression pattern of lncRNAs has not been thoroughly investigated. Questions remain regarding whether MIAT acts concurrently in electrical and structural remodeling, whether its AF regulatory effects are cell-specific, whether both effects can be achieved within the same cell, and if so, whether there is a dynamic ratio of electrical remodeling-related MIAT to structural remodeling-related MIAT during AF progression. Uncovering the spatiotemporal expression patterns of lncRNAs within the multifactorial mechanism of AF development could be a promising avenue for future research. Nevertheless, it is important to acknowledge that discrepancies in results can arise from variations in research methods and models employed across different studies. For example, conflicting results have been reported in studies investigating CACNA1C [32, 43]. Thus, standardizing the criteria for constructing AF animal models will be crucial in future investigations.

The majority of AF models used in animal experiments are induced by AngII, isopropyl, and tachypacing. From a clinical perspective, tachypacing-induced AF in mice appears to be more effective than pharmacological stimulation. However, each model has its advantages and disadvantages, representing different clinical profiles of AF patients. AF and HF are two causally interrelated pathological processes. The incidence of AF increases with age, reflecting the worsening of atrial fibrosis and deterioration of atrial substrate, leading to an increase in abnormal electrical potentials and subsequent AF development. Therefore, each model has its representativeness. AF animal models induced by structural abnormalities, such as ligation of transverse aortic constriction (TAC) and AngII treatment, may be associated with exacerbated atrial fibrosis and impaired atrial substrate. On the other hand, animal models induced by isopropyl and tachypacing, which involve electrical remodeling and neurohormonal abnormalities, are more relevant to younger AF patients with a genetic predisposition. These models better simulate the progression from paroxysmal AF to persistent AF. Most clinical samples or animal models in literature comprise confirmed AF patients, including those with AF-induced HF or requiring surgical intervention. Additionally, the majority of animal models already exhibit phenotypes of atrial fibrosis. However, it is known that the atrial substrate of most paroxysmal AF patients remains relatively preserved, resulting in a higher success rate of radiofrequency ablation. Currently, most animal models in basic research fail to fully mimic the pathological conditions of such patients. This limitation represents a major challenge in AF animal modeling, as it is difficult to simulate AF animals with electrical remodeling but without structural remodeling.

Most of the research conducted on lncRNA differential expression analysis in AF has utilized atrial appendage tissue samples from patients. However, it has been observed that the expression profiles of the left atrial appendage (LAA) and right atrial appendage (RAA) differ from each other [194, 195]. Moreover, the expression profiles of ncRNAs in atrial appendage tissue and peripheral blood samples exhibit even greater disparities. Therefore, it is advisable to confirm the variations in AF-related lncRNA expression patterns between these two sample types. Additionally, it should be noted that many atrial tissue samples used for differential expression analysis were obtained from patients who required open-chest surgery due to other severe complications. These additional pathological factors may also influence RNA expression [196, 197]. In a clinical setting, detecting lncRNAs in blood samples is a more feasible approach for aiding in the diagnosis, prognosis, and analysis of treatment response in AF. Blood-based lncRNA detection offers advantages such as stability, high specificity, and rapid detection through techniques such as PCR. However, the lack of a standardized protocol or standard operating procedures for lncRNA extraction, isolation, and quantification remains a challenge due to the heterogeneity of protocols and quantification platforms.

In recent years, new biomarkers for AF have transitioned from the laboratory to clinical applications [198]. AF-related miRNAs, in particular, have demonstrated potential as noninvasive biomarkers for AF [195, 199]. Based on the current analysis of AF-related lncRNA expression profiles, it is hypothesized that lncRNAs also hold promise as biomarkers for AF. In a recent study, the expression levels of lncRNA PVT1 in the serum of AF patients were examined before and after radiofrequency ablation (RFA). Elevated expression of lncRNA PVT1 was found to be an independent risk factor for recurrence after RFA in AF patients, suggesting that lncRNAs may serve as noninvasive biomarkers or independent predictors of related arrhythmia events [200]. In future studies, it may be beneficial to establish a dynamic monitoring system for serum lncRNA expression levels during the progression from paroxysmal AF to persistent AF to permanent AF. Additionally, comparing serum lncRNA expression levels in patients with paroxysmal AF during both AF rhythm and normal sinus rhythm, or collecting serum samples from patients with initial AF before and after AF onset, could provide valuable insights. Constructing long-term dynamic monitoring patterns of lncRNA expression and establishing a comprehensive database could enhance researchers’ understanding of AF pathogenesis in future basic research on AF.

Indeed, by analyzing clinical samples, common DElncRNAs can be identified in different preclinical studies. For example, the DEmiRNA miR-223-3p has been predicted as a potential upstream regulatory factor by bioinformatics tools, and KCNQ1OT1 has been identified as a candidate upstream regulator. This lncRNA has been previously found to be differentially expressed in AF animal models and demonstrated to play a regulatory role in AER. Additionally, DElncRNAs such as MIAT and GAS5 have shown differential expression in various clinical samples or animal models, indicating the ability of bioinformatics tools to identify meaningful DElncRNAs. These DElncRNAs hold potential for development as biomarkers and therapeutic targets. However, introducing lncRNAs into clinical practice as biomarkers still presents certain challenges. Preclinical studies have suggested that the lncRNA PVT1 could serve as a marker for AF recurrence after radiofrequency ablation. However, extensive basic research is still required to validate whether PVT1 acts as an upstream regulatory molecule triggering AF recurrence. It is also unclear whether PVT1 can serve as a therapeutic target in clinical settings, despite its identification as an upstream regulatory molecule leading to atrial fibrosis in two other AF studies [38, 48].

### LncRNA-based approaches

First, ncRNA-based drugs offer the advantage of precise targeting to specific genes or genomic regions, enabling highly specific regulation and reducing non-specific side effects. Additionally, ncRNA drugs can target the non-coding regions of the genome, which still hold significant exploration potential for discovering new therapeutic targets. In our review, we discussed the AF regulatory network involving lncRNA-miRNA-mRNA interactions. By targeting upstream lncRNAs, it may be possible to better inhibit the pathophysiological processes underlying AF, particularly by targeting the same lncRNA with different downstream effects. However, the immunogenicity of ncRNA drugs and the challenges in delivery remain major obstacles. Some ncRNA drugs may induce immune system reactions, resulting in immunogenic and inflammatory responses. Moreover, the delivery of ncRNA drugs to target cells or tissues requires overcoming biological barriers and addressing stability issues. The development of appropriate delivery systems and technologies is crucial to ensure the effectiveness of these drugs. Furthermore, determining the correct dosage and treatment timing during the delivery process is critical for the efficacy of ncRNA drugs. This involves a thorough understanding of the therapeutic targets and monitoring and adjusting treatment responses, which necessitates extensive foundational research for validation.

In the context of downregulating lncRNA expression, small interfering RNAs (siRNAs) represent a direct approach that can be employed for lncRNA targeting. siRNAs function by recruiting the RNA-induced silencing complex (RISC) to induce degradation of the targeted lncRNA. Within the aforementioned literature review, it is evident that numerous preclinical models have employed siRNA delivery to interfere with lncRNA expression and study the role of lncRNAs in AF [33, 40]. This demonstrates the systematic ability of siRNAs to target specific lncRNAs. However, siRNA still faces a major challenge: off-target effects. Although this can be mitigated through lower doses and modifications of siRNAs or delivery systems, off-target effects have not been completely resolved. While siRNA drugs targeting mRNAs have already been approved, siRNA therapy based on ncRNAs is still under exploration. Promising progress has been made with miRNAs entering clinical trial stages for cardiovascular diseases, which signifies a new era in the research of siRNA drugs based on lncRNAs [201].

Antisense oligonucleotides (ASOs) are a class of chemically synthesized short single-stranded oligonucleotides with DNA sequences consisting of 15 to 25 nucleotides. They can bind to complementary RNA molecules and recruit RNAse H, leading to RNA degradation and alteration of downstream protein expression. The chemical modifications of ASOs have overcome the limitations of low cellular permeability and poor stability, resulting in significant progress in improving their pharmacological properties. The latest generation of ASOs, such as those modified with locked nucleic acid (LNA), exhibit enhanced affinity and stability. However, they still have certain off-target effects, and their delivery is currently limited to injection, lacking the option for oral administration [202, 203]. Additionally, studies have shown that lncRNAs can be enriched in both the cytoplasm and the nucleus of cells [204, 205]. siRNAs and shRNAs are ineffective in targeting lncRNAs in the cell nucleus. However, ASOs can specifically target lncRNAs in the nucleus, complementing the effects of siRNAs [206]. This has led to the emergence of a hybrid knockdown approach that combines ASOs and RNA interference, enabling simultaneous downregulation of lncRNAs in both the cytoplasm and the nucleus. Traditionally, antisense therapy was primarily aimed at upregulating gene expression, with several lncRNAs acting as natural antisense transcripts (NATs) of target genes. Based on our review of AF-related lncRNA-miRNA-mRNA pathways, most lncRNAs upregulate target genes by attenuating the inhibitory effects of miRNAs on downstream mRNAs through the sponge effect. Therefore, it is conceivable that ASO therapy can target upstream lncRNAs that promote AF, effectively downregulating harmful lncRNAs and further suppressing target mRNA expression, potentially inhibiting the occurrence and progression of AF. This study validates the effectiveness of ASO therapy in disrupting the targeted lncRNAs and suppressing downstream target gene expression when ASOs act as ceRNAs [207]. However, the progress of ASO-based lncRNA therapy for cardiovascular diseases remains limited. Only one preclinical study has demonstrated the potential of ASOs targeting lncRNA cardiac hypertrophy-associated transcript (Chast) in preventing and attenuating TAC-induced pathological cardiac remodeling in mice [208]. Nonetheless, this encourages further research on ASO-based lncRNAs, and we anticipate more studies in the future.

### Delivery system

Currently, there are numerous preclinical models utilizing virus-based vectors for lncRNA research [33, 209]. Among these vectors, lentiviral vectors are the most widely used for delivering lncRNA. They exhibit high transduction efficiency and long-term expression. As a typical tool, lentiviral vectors are employed in many studies to interfere with lncRNA by loading specific lncRNA or siRNA, enabling targeted overexpression or knockdown of lncRNA [210]. Adenoviral vectors are also commonly used for lncRNA overexpression or interference [125, 209]. For instance, an effective knockdown model of the Mirf gene can be established by injecting adenoviral vectors carrying Mirf shRNA into mice [209]. These virus-based vector systems possess certain advantages, including efficient gene delivery: they are highly effective in transferring lncRNA into cardiac cells, resulting in high-level expression. Furthermore, certain virus-based vector systems can achieve stable long-term gene expression, ensuring the sustained presence and function of lncRNA. However, they still exhibit immunogenicity, as some virus-based vectors may activate the immune system, leading to immune and inflammatory responses. This limitation may restrict their application and have adverse effects on long-term expression. Numerous studies have utilized virus-based vector systems in animal models to validate lncRNA silencing using virus-based vector-mediated shRNA expression in in vivo experiments. However, most clinical trials based on virus-based vectors are currently in Phase I or Phase II. Only one trial, focusing on the treatment of HF through overexpression of sarcoplasmic reticulum Ca^2+^-ATPase (SERCA2a) pump, has reached Phase IIb and represents the first and only AAV clinical trial in the cardiovascular field [211]. In summary, considering the safety concerns associated with virus-based vectors in systemic administration, they have gradually been replaced by other vectors. In the following discussion, we will explore the more widely used lipid-based or nanoparticle-based delivery systems. Liposomes possess numerous desirable properties. In addition to reducing toxicity and immunogenicity, liposomes encapsulation enhances drug stability. Liposomes are ideal materials for targeted delivery of various types of RNA, considering the advancements in nanomaterials, lipid nanoparticles (LNPs), are becoming increasingly popular in preclinical trials exploring the functional and therapeutic potential of RNA. Among them, LNPs-based mRNA intracellular delivery systems have entered numerous large clinical trials, particularly following significant progress in COVID-19 vaccine development. The key distinction between LNPs and conventional liposomes lies in their micellar structures within the particle core [212], providing better stability and reducing the likelihood of RNA degradation. Moreover, LNPs typically have smaller particle sizes, facilitating easier penetration of cell membranes and enhanced intracellular delivery efficiency, thereby increasing the biological activity of RNA. Importantly, the composition of lipid nanoparticles reduces the presence of permanent charges, mitigating high cytotoxicity associated with carrier systems. By stabilizing the membrane structure of nanomaterials, it also increases the chances of escape scape from the endosome and lowers the risk of immunogenic reactions, thus improving their safety in clinical applications [213]. Apart from the successful local delivery of LNP-mRNA vaccines, LNPs enable targeted delivery to organs through systemic administration [214]. Additionally, due to the challenge of targeting highly specific nuclear lncRNA with nuclear envelope protection, combining LNPs with ASOs provides a promising approach. LNP-ASO combination allows for targeted delivery of nuclear lncRNA with good compatibility and immunogenicity. For instance, in a lung cancer xenograft animal model, LNP-encapsulated ASOs targeting nuclear lncRNA MALAT1 demonstrated significant inhibition of tumor metastasis and notable therapeutic efficacy [215]. These studies have shown potential for therapeutic approaches based on lncRNA. Although clinical trials targeting lncRNA using LNPs have not yet commenced, in vitro and in vivo preclinical experiments involving lncRNA delivery frequently employ LNPs [216, 217]. Moreover, a preclinical study in the cardiovascular field revealed the effective suppression of fibroblast-induced fibrogenic differentiation by intrapericardial injection of LNP-encapsulated lncRNA-Tcf21 antisense RNA inducing demethylation (TARID), leading to improved cardiac function and electrophysiological properties in mouse and pig models, establishing the therapeutic potential of LNP-TARID for myocardial fibrosis [218]. Based on our comprehensive review of lncRNA-mediated pathological processes in AF, it has been observed that certain protective lncRNAs are downregulated during AF pathogenesis. We believe that the targeted delivery of lncRNA using LNP encapsulation, through systemic administration or local injection, can potentially modulate lncRNA expression and inhibit the occurrence and development of AF.

Additionally, different species can benefit from a variety of administration routes for ncRNA-based drugs. For instance, rabbits and dogs with AF can receive intracardiac injections, while pigs commonly undergo intravenous injections, catheter-based injections, coronary artery injections, or subcutaneous injections. Currently, preclinical studies in cardiac disease models employ local administration methods such as intracoronary or intramyocardial injections. However, the translation of these approaches into clinical practice poses uncertainties and challenges. In clinical settings, subcutaneous injections, intravenous injections, and intradermal injections are more favorable and easily implemented delivery routes in humans. Consequently, achieving effective systemic delivery necessitates improved targeting specificity and tissue selectivity. In the context of cardiovascular diseases, the successful delivery of drugs to cardiomyocytes, endothelial cells, CFs, or immune cells within the heart may hold paramount importance, depending on the underlying pathological mechanisms of specific clinical conditions.

### Perspective and challenge of exosomal lncRNAs

Exosomes have gained significant attention in recent years as a promising area of research for targeted therapeutics. There has been abundant investigation into the use of exosomal ncRNAs in cardiovascular diseases, including coronary arteriosclerosis, MI, and pulmonary hypertension [219–222]. Although numerous studies have identified exosomal DEmiRNAs in the serum of AF patients [223], research on AF-related exosomal lncRNAs remains sporadic. However, a recent study successfully identified specific exosomal lncRNAs in the serum of persistent AF patients. Differential expression analysis revealed a strong correlation between the expression level of serum exosomal lncRNA LOC107986997 and persistent AF, suggesting its potential as a diagnostic biomarker for AF [224]. Furthermore, comparative analysis with other relevant studies demonstrated that differentially expressed exosomal lncRNA LINC00636 was found in the pericardial fluid (PF) of AF patients. This particular lncRNA was shown to influence the proliferation of CFs and atrial fibrosis by regulating miR-450a-2-3p [225]. Studies focusing on PF may provide greater specificity in identifying AF-related DElncRNAs compared to blood-based studies. Additionally, the previously mentioned MIAT was validated in a mouse model, where it was found to promote AF progression through serum EV-derived regulation [37]. In contrast, XIST in EVs from AMSCs was found to ameliorate AF by attenuating cardiomyocyte pyroptosis through counteracting the inhibitory effect of miR214-3p on Arl2 [49]. These findings indicate that these lncRNAs can be delivered and regulate the development of AF through EVs. Notably, EVs secreted by MSCs possess certain cardiac repair functions, which highlights the significance of exosomal lncRNAs secreted by MSCs in attenuating AF progression and provides new potential applications for exosomal lncRNAs in AF [226]. In essence, EVs are membrane structures that facilitate the transportation of DNA, RNA, and proteins secreted by various cells into the extracellular space. They play a prominent role in cell-to-cell communication [227]. The advantages of EVs containing lncRNAs include low immunogenicity, high specificity, and the ability to protect internal cargo from degradation by RNases. These features make the delivery of lncRNA-containing EVs a potential therapeutic strategy. In recent years, technologies for extracting and isolating EVs from blood or other body fluids have flourished. However, the clinical evaluation of exosomal lncRNAs as diagnostic and therapeutic targets is still lacking. In the future, it is expected that AF-related exosomal lncRNAs will gradually transition from the laboratory to clinical applications, paving the way for their use in the diagnosis and treatment of AF.

## Conclusion

**Fig. 2:**
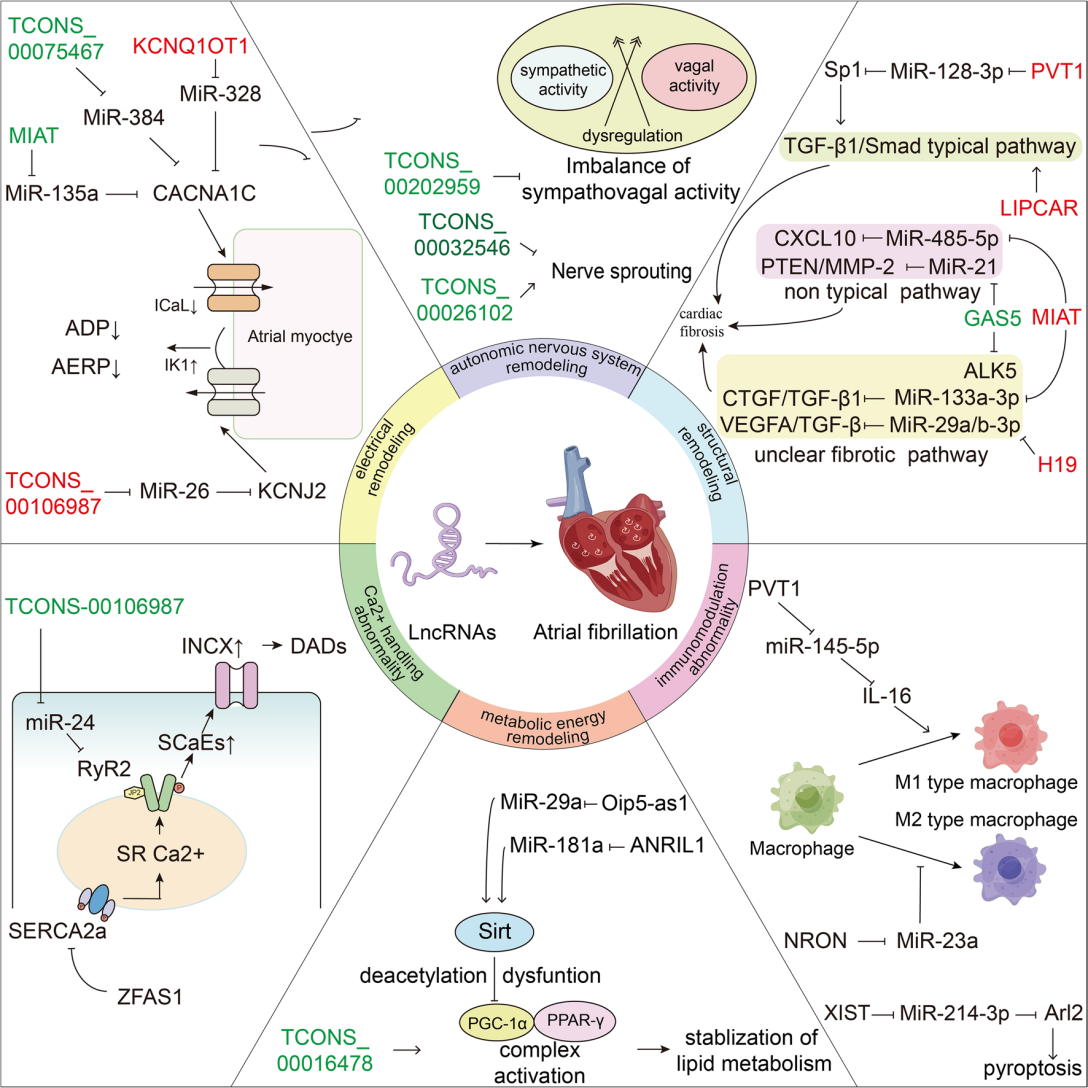
LncRNAs involved in AF pathophysiology. LncRNAs contribute to each of six pathophysiological processes of AF. These AF-related lncRNAs and their molecular parterners or downstream targets are shown for electrical remodeling, structural remodeling, Ca^2+^ handling abnormality, autonomic nervous system remodeling, energy metabolic remodeling and immunoregulation abnormality. Within the context of this figure, the lncRNAs highlighted in green represent a downregulation, whereas those in red indicate an upregulation. It is important to note that these aberrant expression patterns were specifically observed in samples known to exhibit atrial fibrillation.. Abbreviation: ALK5, The TGFβ type I receptor kinase; ANRIL, Antisense nelayed afterdepolarization; I_CaL_, L-type calcium current; I_K1_, Inward-rectifying K + current; INCX, Na^+^/Ca^2+^ exchanger (NCX) current; on-coding RNA at the INK4 locus; APD, Action potential duration; AERP, Atrial effective refractory period; Arl2, ADP ribosylation factor like GTPase 2; CTGF, Connective tissue growth factor; CACNA1C, α1c subunit of the calcium channel; CXCL10, C-X-C motif chemokine ligand 10; DAD, DKCNJ2, Potassium inwardly rectifying channel subfamily J member 2; KCNQ1OT1, Potassium voltage-gated channel subfamily Q member 1 opposite strand 1; MIAT, Myocardial infarction associated transcript; MMP2, Matrix metallopeptidase2; NRON, Non-coding repressor of NFAT; OIP5‐AS1, Opa-interacting protein 5-antisense transcript 1; PVT1, Plasmacytoma variant translocation 1; PPARs, Peroxisome proliferator-activated receptors; PGC-1 s, PPARγ coactivator-1 s; PTEN, phosphatase and tensin homolog; RyR2, Ryanodine receptor 2; SR, Sarcoplasmic reticulum; SERCA2, Sarcoplasmic/endoplasmic reticulum Ca^2+^ ATPase 2; SCaEs, SR Ca^2+^-release events; Sp1, Specificity protein1; TGF-β, Transforming growth factor type-β; VEGFA, Vascular endothelial growth factor A; XIST, X-inactive specific transcript; ZFAS1, Zinc finger antisense 1

In recent years, a growing body of evidence has demonstrated the crucial role of lncRNAs as key regulators in the onset and maintenance of AF. Particularly, they function as upstream molecules that modulate the expression of miRNAs and mRNAs, thus influencing key remodeling pathways in AF’s pathophysiology (Fig. 2). The interplay between lncRNA-miRNA-mRNA interactions and downstream signaling pathways is involved in various pathophysiological mechanisms of AF, including electrical remodeling, structural remodeling, abnormal Ca^2+^ handling, autonomic nervous system remodeling, energy metabolic remodeling, and immunomodulation abnormalities. This review provides a comprehensive summary of AF-related lncRNAs and their associated downstream pathways. These lncRNAs hold promising potential as novel biomarkers for AF, and their identification represents a new class of therapeutic targets for halting or reversing the progression of AF. However, it is essential not only to further dissect the interaction network of AF-related lncRNA-miRNA-mRNA but also to elucidate the specific molecular mechanisms involved. In addition to considering the role of lncRNAs as ceRNAs, a deeper understanding of their precise molecular functions is necessary. Furthermore, it is crucial to optimize bioinformatics methodologies, standardize the sampling and detection of biospecimens, and establish well-defined AF animal models. Exosomal lncRNAs, with their advantages of low immunogenicity, high specificity, and efficient delivery, hold great potential in the diagnosis, prognosis, and targeted therapy of AF. Nevertheless, to fully comprehend the biogenesis and develop targeted therapeutics for lncRNAs in AF, continuous refinement of bioinformatics tools and biomaterials is required to address existing challenges in biotherapeutics. As research on AF-related lncRNAs advances, new and exciting approaches for the diagnosis and treatment of AF will emerge. The integration of interdisciplinary efforts involving bioinformatics, biomaterials, and molecular biology will be instrumental in unraveling the full potential of lncRNAs in AF and paving the way for innovative therapeutic strategies.

## Data Availability

The figure was created by Figdraw (http://www.figdraw.com).
